# Dose–response association between metabolic syndrome component count and metabolic dysfunction-associated fatty liver disease, and independent graded association of visceral fat area: a cross-sectional study in a health-checkup population

**DOI:** 10.3389/fpubh.2026.1842607

**Published:** 2026-06-16

**Authors:** Qian Xie, Hu Li, Xiaotong Hu, Jie Wan, Xia Gao, Liang Huang, Yan Long, Xia Xu, Chunmao Jiang

**Affiliations:** Department of Special Medicine, Daping Hospital, Army Medical University, Chongqing, China

**Keywords:** health screening, metabolic dysfunction-associated fatty liver disease, metabolic syndrome, risk stratification, visceral fat area

## Abstract

**Objectives:**

To quantify the dose–response association between metabolic syndrome (MetS) component count and metabolic dysfunction-associated fatty liver disease (MAFLD), and to evaluate the independent graded association of visceral fat area (VFA) with MAFLD.

**Materials and methods:**

This single-center cross-sectional study enrolled adults (≥18 years) undergoing routine health checkups from July 2024 to December 2025. MAFLD was defined according to the Chinese Guideline for the Prevention and Treatment of Metabolic Dysfunction-associated Fatty Liver Disease (Version 2024), based on imaging-confirmed fatty liver plus predefined metabolic criteria after exclusion of excessive alcohol consumption and other specific causes of fatty liver. MetS component count was categorized as 0, 1, 2, or ≥3, and VFA, measured by bioelectrical impedance, was analyzed in quartiles. Multivariable logistic regression estimated ORs (95% CI) using hierarchical models. Analyses were also performed in the normal-BMI subgroup, with discriminatory ability assessed by the area under the curve (AUC).

**Results:**

Among 7,192 participants (mean age 42.7 years; 51.9% men), 2,794 (38.8%) were classified as having MAFLD. MAFLD prevalence increased markedly with increasing metabolic syndrome (MetS) component count (0/1/2/≥3: 14.0, 25.8, 55.8, and 79.7%, respectively; *P* for trend < 0.001). In Model 2, compared with 0 components, the ORs (95% CIs) for MAFLD were 2.75 (2.22–3.41), 5.33 (4.24–6.71), and 10.26 (7.88–13.43) for 1, 2, and ≥3 components, respectively. In Model 3, which additionally included VFA quartiles, the association remained significant, with corresponding ORs (95% CIs) of 2.56 (2.06–3.18), 4.78 (3.79–6.04), and 9.23 (7.03–12.13). VFA also showed an independent graded association with MAFLD (Q4 vs. Q1: OR 3.27, 95% CI 2.44–4.39). Similar patterns were observed in the normal-BMI subgroup, where higher MetS component count remained strongly associated with MAFLD and VFA retained an independent graded association (Q4 vs. Q1: OR 3.67, 95% CI 2.12–6.36). The fully adjusted model showed good discrimination overall (AUC 0.887, 95% CI 0.879–0.895).

**Conclusion:**

MetS component count showed a clear dose–response association with MAFLD, while VFA showed an independent graded association. These findings support the integration of cumulative metabolic burden and visceral adiposity into MAFLD-oriented risk stratification in routine health screening.

## Introduction

Metabolic dysfunction–associated fatty liver disease (MAFLD) is a chronic liver disease characterized by hepatic fat accumulation against a background of metabolic dysfunction. It has become the most common chronic liver disease worldwide, and its prevalence continues to rise, placing a substantial and growing burden on public health (1, 2). As obesity, type 2 diabetes, and related cardiometabolic disorders become increasingly common, the population-level impact of MAFLD continues to intensify. Consequently, early identification of high-risk individuals using tools suitable for routine screening has become a clinical and public health priority (3–5).

Metabolic syndrome (MetS)—a clinical cluster of central obesity, hyperglycemia, dyslipidemia, and elevated blood pressure—is recognized as one of the core determinants of MAFLD development and progression (6–8). Traditionally, MetS has been operationalized using a simple component-count approach. However, this dichotomous framework is inherently reductive and may fail to capture the cumulative metabolic burden and its graded association with MAFLD risk. Consequently, attention has shifted toward MetS component count as a direct, pragmatic index of cumulative metabolic dysregulation—and thus a more granular, graded measure of metabolic risk (9, 10). Nevertheless, whether MetS component count demonstrates a clear dose–response relationship with MAFLD—and thus provides a graded risk metric for MAFLD-oriented risk stratification—remains incompletely characterized, particularly in large, real-world screening populations where subclinical metabolic abnormalities are common.

In addition to MetS component count, visceral adiposity is an important contributor to MAFLD (11, 12). Visceral fat area (VFA), a quantitative marker of visceral fat accumulation, is routinely obtained in health-checkup settings and may capture metabolic risk and ectopic fat deposition more precisely than body mass index (BMI) (13). Although VFA has been consistently linked to MAFLD-related outcomes, evidence remains limited on the independent and joint associations of MetS component count and VFA with MAFLD and on whether VFA shows an independent graded association in models already incorporating MetS component count. This issue is especially pertinent for normal-weight individuals, in whom metabolically driven liver disease may be under-recognized by BMI-based assessment.

Leveraging the large sample size and screening-oriented setting of a health-checkup population, we conducted a single-center cross-sectional study to quantify associations of MetS component count and MAFLD, with particular attention to the dose–response pattern across increasing component categories. We also examined whether VFA, as a quantitative marker of visceral adiposity, showed an independent graded association with MAFLD in MetS component count–based models, including among individuals with normal BMI. In addition, we further explored the discriminative performance of VFA in comparison with waist circumference and waist-to-hip ratio in the normal-BMI subgroup. By integrating cumulative metabolic burden and visceral adiposity, this study aimed to support MAFLD-oriented risk assessment in routine health screening.

## Methods

### Study design and population

**Figure 1:**
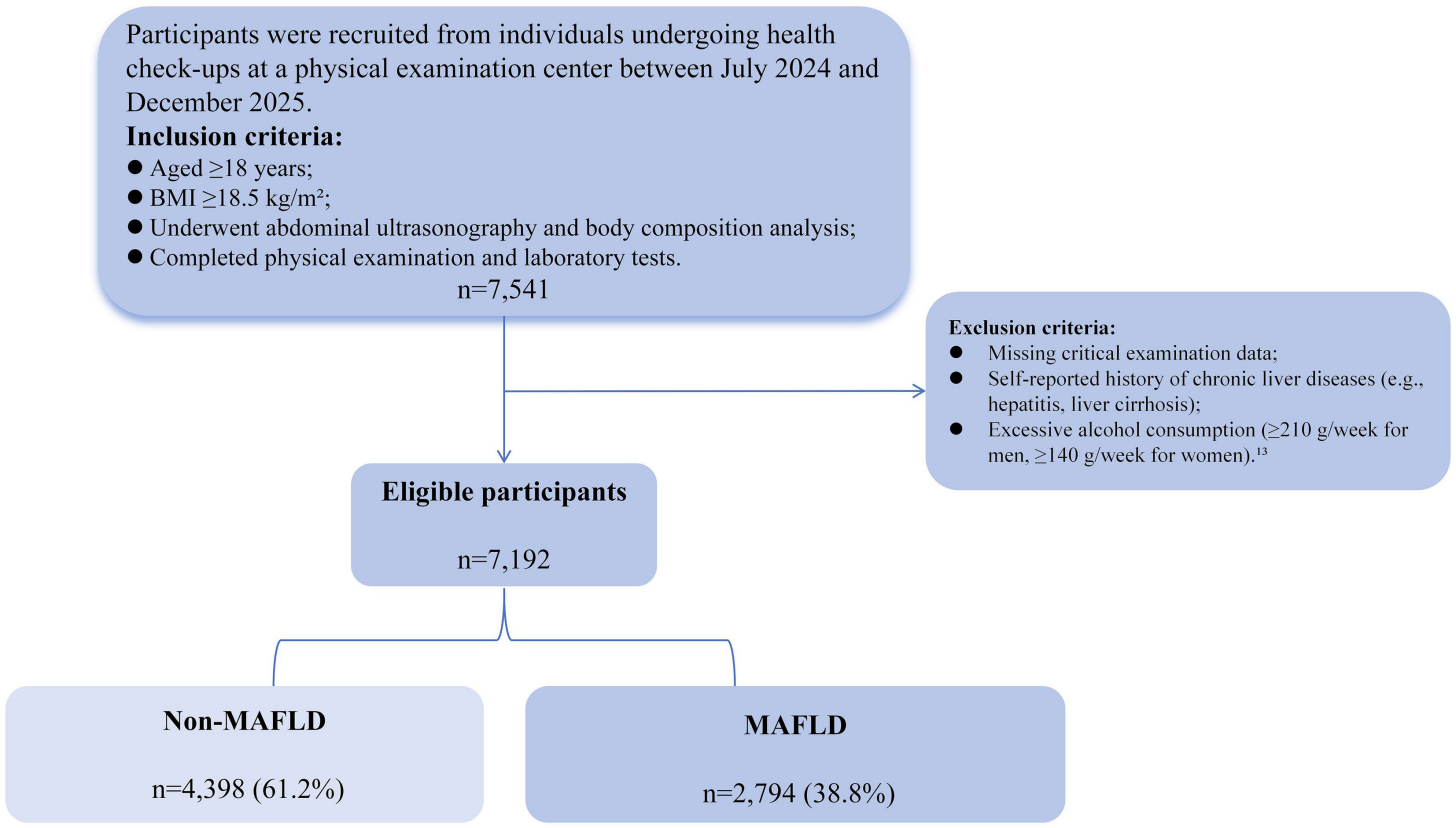
Flow diagram of participant selection.

This was a single-center cross-sectional study. We included consecutive adults (≥18 years) who underwent routine health examinations at a health-checkup center between July 2024 and December 2025. Inclusion criteria were: (1) age ≥18 years; (2) body mass index (BMI) ≥ 18.5 kg/m^2^; and (3) completion of abdominal ultrasonography and body composition assessment, with complete anthropometric and laboratory data available. Exclusion criteria were: (1) substantial missing data; (2) a known history of other chronic liver diseases (e.g., viral hepatitis or cirrhosis); and (3) excessive alcohol consumption, defined as ≥210 g/week for men and ≥140 g/week for women (14, 15). In total, 7,192 participants were included in the final analysis. Among them, 2,834 had imaging-detected fatty liver. After applying the Chinese Guideline for the Prevention and Treatment of Metabolic Dysfunction-associated Fatty Liver Disease (Version 2024) (15), 2,794 participants were classified as having MAFLD and 4,398 as not having MAFLD (Figure 1).

### Metabolic syndrome component count

Metabolic syndrome (MetS) components were defined using Chinese-specific cutoffs (16):

- Central obesity: waist circumference ≥90 cm in men or ≥85 cm in women;
- Hypertriglyceridemia: triglycerides ≥1.7 mmol/L;
- Low HDL-C: HDL-C < 1.04 mmol/L;
- Elevated blood pressure: systolic blood pressure≥130 mmHg and/or diastolic blood pressure ≥85 mmHg, or a prior diagnosis of hypertension with current treatment;
- Elevated fasting glucose: fasting plasma glucose≥6.1 mmol/L (participants with random glucose measurements were excluded), or a prior diagnosis of diabetes with current treatment.

MetS component count was calculated as the number of positive components (range, 0–5) and categorized as 0, 1, 2, or ≥3 components (≥3 defining MetS). These criteria were used to operationalize the exposure variable MetS component count in the main analyses, whereas MAFLD was defined separately according to the Chinese guideline, as described below.

### Outcome: metabolic dysfunction-associated fatty liver disease

MAFLD was defined according to the Chinese Guideline for the Prevention and Treatment of Metabolic Dysfunction-associated Fatty Liver Disease (Version 2024) (15). Hepatic steatosis was identified by abdominal ultrasonography performed by certified sonographers following a standardized protocol. Fatty liver was defined by typical imaging features, including diffusely increased hepatic echogenicity (“bright liver”), increased hepatorenal contrast, and blurring/poor visualization of intrahepatic vascular structures and the diaphragm (17).

Participants with imaging-confirmed fatty liver were classified as having MAFLD if they had any of the following predefined metabolic abnormalities after exclusion of excessive alcohol consumption (≥210 g/week for men and ≥140 g/week for women) and other specific causes of fatty liver (15):

- Overweight/obesity, defined as BMI ≥ 24.0 kg/m^2^, or waist circumference ≥90 cm in men or ≥85 cm in women, or excessive body fat content and percentage;
- Blood pressure ≥130/85 mmHg, or undergoing antihypertensive medication therapy;
- Dysglycaemia or type 2 diabetes mellitus, defined as fasting plasma glucose ≥6.1 mmol/L, or 2-h postprandial plasma glucose ≥7.8 mmol/L, or HbA1c ≥ 5.7%, or history of type 2 diabetes mellitus, or HOMA-IR ≥ 2.5;
- Plasma triglycerides ≥1.70 mmol/L, or undergoing lipid-lowering medication therapy;
- HDL-cholesterol ≤1.0 mmol/L in men and ≤1.3 mmol/L in women, or undergoing lipid-lowering medication therapy.

Because the Chinese MAFLD guideline does not specify a numeric cutoff for excessive body fat percentage, we operationalized this criterion using the Chinese Guidelines for the Clinical Management of Obesity (2024 Edition), defining excessive body fat as body fat percentage >25% in men and >30% in women (18). Based on this diagnostic framework, 2,794 of 2,834 participants with imaging-detected fatty liver met the criteria for MAFLD and were included in the MAFLD group.

### Visceral fat area measurement

Visceral fat area (VFA) was measured using bioelectrical impedance analysis (BIA) with a body composition analyzer (InBody 770; InBody Co., Ltd., Seoul, Republic of Korea) according to the manufacturer’s standardized protocol. VFA was recorded in cm^2^ and analyzed in quartiles. Body fat percentage was obtained from the same body composition assessment.

### Anthropometric and laboratory measurements

Anthropometric measurements and blood pressure were obtained during the routine health examination according to standardized procedures. Fasting venous blood samples were collected after an overnight fast, and fasting plasma glucose, HbA1c, serum lipids, liver function tests, uric acid, homocysteine, and other routinely collected biochemical indices were measured in the health-checkup laboratory using routine assays.

### Statistical analysis

All analyses were performed using SPSS Statistics (version 27.0). Continuous variables are presented as mean ± standard deviation (SD) or median (interquartile range [IQR]), as appropriate, and categorical variables as number (%). Between-group comparisons used the independent-samples *t* test or Mann–Whitney *U* test for continuous variables and the *χ*^2^ test for categorical variables, as appropriate.

Multivariable logistic regression was performed to estimate odds ratios (ORs) and 95% confidence intervals (CIs) for MAFLD.

To evaluate the independent associations of individual MetS components with MAFLD, we fitted a multivariable logistic regression model in the total population including sex, age, central obesity, hyperglycemia, elevated blood pressure, hypertriglyceridemia, and low HDL-C entered simultaneously. Sex-stratified models were further fitted in men and women separately. The relative contribution of each variable was assessed using the Wald *χ*^2^ statistic, with larger values indicating greater contribution to the model.

To further assess whether the associations of MetS components with MAFLD differed by sex, interaction analyses were performed by adding interaction terms between sex and each MetS component, as well as sex and age, into a multivariable logistic regression model adjusted for all main effects.

To examine the associations of MetS component count and visceral fat area (VFA) with MAFLD, we fitted a series of hierarchical models (Tables 1, 2): Model 1 (unadjusted); Model 2 (adjusted for age, sex, and body mass index [BMI]); and Model 3 (Model 2 plus VFA quartiles [Q1–Q4] defined in the overall sample). The same modeling strategy was applied in the normal-BMI subgroup (defined as BMI 18.5–23.9 kg/m^2^ according to Chinese criteria for adults) as a prespecified subgroup analysis (Table 2) (19).

**Table 1:** Associations of MetS component count and VFA with MAFLD.

Predictor	Model 1, OR (95% CI)	Model 2, OR (95% CI)^*^	Model 3, OR (95% CI)^*†^
MetS component count (ref = 0)			
1 component	2.216(1.769–2.554)	2.754 (2.224–3.410)	2.558 (2.059–3.177)
2 components	7.715 (6.366–9.350)	5.334 (4.243–6.705)	4.784 (3.791–6.036)
≥3 components	23.996 (19.200–29.990)	10.264 (7.884–13.432)	9.232 (7.025–12.132)
VFA quartiles (ref = Q1)			
Q2			1.894 (1.521–2.360)
Q3			2.440 (1.919–3.104)
Q4			3.268 (2.436–4.386)

Outcome was MAFLD. ^*^Model 2 was adjusted for age (continuous), sex, and BMI. ^†^Model 3 was additionally adjusted for VFA quartiles (overall Q1–Q4).

MAFLD, metabolic dysfunction–associated fatty liver disease; MetS, metabolic syndrome; VFA, visceral fat area; BMI, body mass index; OR, odds ratio; CI, confidence interval.

**Table 2:** Associations of MetS component count and VFA with MAFLD in the normal-BMI subgroup (BMI 18.5–23.9 kg/m^2^; *n* = 3,329).

Predictor	Model 1, OR (95% CI)	Model 2, OR (95% CI)^*^	Model 3, OR (95% CI)^*†^
MetS component count (ref = 0)			
1 component	3.137 (2.073–4.747)	5.986 (3.846–9.317)	5.605 (3.590–8.751)
2 components	9.349 (6.021–14.516)	12.515 (7.757–20.191)	11.371 (7.018–18.424)
≥3 components	18.411 (10.885–31.140)	22.485 (12.435–40.660)	21.269 (11.684–38.717)
VFA quartiles (ref = Q1)			
Q2			2.096 (1.521–2.889)
Q3			2.618 (1.753–3.912)
Q4			3.668 (2.115–6.362)

Outcome was MAFLD in the normal-BMI subgroup (BMI 18.5–23.9 kg/m^2^, defined according to Chinese criteria for adults) (19). ^*^Model 2 was adjusted for age (continuous), sex, and BMI. ^†^Model 3 was additionally adjusted for VFA quartiles (overall Q1–Q4).

MAFLD, metabolic dysfunction–associated fatty liver disease; MetS, metabolic syndrome; VFA, visceral fat area; BMI, body mass index; OR, odds ratio; CI, confidence interval.

Discriminative ability was assessed using the area under the receiver operating characteristic curve (AUC), reported with 95% CIs. For the primary model, AUC was calculated for the fully adjusted model incorporating MetS component count and VFA. In supplementary analyses, additional models combining MetS component count with continuous VFA, waist circumference (WC), or waist-to-hip ratio (WHR) were evaluated by ROC analysis in the overall population after adjustment for age, sex, and BMI. In the normal-BMI subgroup, additional ROC analyses were performed to compare the discriminative ability of continuous VFA, WC, and WHR for MAFLD after adjustment for age, sex, and BMI. Pairwise comparisons of AUCs using the DeLong test were conducted for VFA, WC, and WHR in the normal-BMI subgroup.

All tests were two-sided, and *p* < 0.05 was considered statistically significant.

## Results

Among 7,192 eligible participants included in the final analysis, 2,794 (38.8%) were classified as having MAFLD. The study population comprised 3,734 men and 3,458 women, with a mean age of 42.65 ± 12.38 years. The sex-specific prevalence of MAFLD was 52.7% in men (1,968/3,734) and 23.9% in women (826/3,458), with a significantly higher prevalence in men (*p* < 0.001). Metabolic syndrome, defined as ≥3 MetS components, was present in 14.6% of participants (1,048/7,192). Compared with non- MAFLD participants, those with MAFLD were more likely to be male and had higher age, BMI, body fat percentage, visceral fat area, waist circumference, and waist-to-hip ratio, systolic and diastolic blood pressure, total cholesterol, triglycerides, LDL-C, fasting plasma glucose, HbA1c, ALT, AST, uric acid, and homocysteine, whereas HDL-C was lower (all *p* < 0.05; Table 3). Carotid plaque was also more frequent in the MAFLD group (38.4% vs. 31.6%, *p* = 0.003).

**Table 3:** Baseline characteristics of participants by MAFLD status.

Characteristic	Non-MAFLD (*n* = 4,398)	MAFLD (*n* = 2,794)	Test statistic	*p* value
Sex, male, *n* (%)	1,766 (40.2)	1,968 (70.4)	627.628	<0.001
Age, years	39 (31, 52)	46 (35, 54)	−12.670	<0.001
BMI, kg/m^2^	22.83 (21.11, 24.61)	26.73 (25.01, 28.63)	−51.164	<0.001
Body fat percentage, %	29.00 (24.20, 33.60)	31.75 (27.50, 36.30)	−18.234	<0.001
Visceral fat area, cm^2^	75.00 (60.40, 95.50)	105.70 (85.10, 131.15)	−38.191	<0.001
Waist circumference, cm	77.0 (72.0, 83.0)	90.0 (85.0, 95.0)	−50.644	<0.001
Waist-to-hip ratio	0.88 (0.85, 0.91)	0.94 (0.91, 0.97)	−44.294	<0.001
Systolic blood pressure, mmHg	111 (101, 122)	122 (111, 134)	−26.803	<0.001
Diastolic blood pressure, mmHg	69 (62, 76)	76 (68, 84)	−26.360	<0.001
Total cholesterol, mmol/L	4.85 (4.27, 5.51)	5.20 (4.58, 5.87)	−14.508	<0.001
Triglycerides, mmol/L	1.02 (0.75, 1.43)	1.87 (1.32, 2.76)	−42.308	<0.001
LDL-C, mmol/L	2.89 (2.43, 3.39)	3.24 (2.79, 3.76)	−19.485	<0.001
HDL-C, mmol/L	1.52 (1.31, 1.74)	1.29 (1.11, 1.47)	−29.851	<0.001
Fasting plasma glucose, mmol/L	4.79 (4.50, 5.13)	5.10 (4.70, 5.63)	−21.747	<0.001
HbA1c, %	5.6 (5.4, 5.9)	5.9 (5.6, 6.4)	−7.576	<0.001
ALT, U/L	16.7 (12.4, 23.8)	29.1 (20.2, 43.7)	−37.929	<0.001
AST, U/L	20.7 (17.9, 24.9)	24.4 (20.5, 30.7)	−23.906	<0.001
Uric acid, μmol/L	298.50 (252.50, 358.35)	382.60 (318.00, 446.75)	−33.715	<0.001
Homocysteine, μmol/L	11.55 (9.30, 14.60)	12.40 (9.85, 15.00)	−2.002	0.045
Carotid plaque, *n*/*N*(%)^*^	272/861 (31.6)	316/822 (38.4)	8.685	0.003
MetS component count, *n* (%)			1,443.702	<0.001
0 components	1,010 (24.3)	165 (6.2)		
1 component	2,229 (53.70)	774 (29.2)		
2 components	699 (16.8)	881 (33.2)		
≥3 components(MetS)	213 (5.1)	835 (31.5)		

Data are presented as mean ± SD, median (IQR), or *n* (%), as appropriate. *p* values were obtained using the independent-samples *t* test, Mann–Whitney *U* test, or *χ*^2^ test, as appropriate.

^*^Carotid plaque was assessed in a subset of participants who underwent carotid ultrasonography (*n* = 1,683; Non-MAFLD, *n* = 861; MAFLD, *n* = 822); percentages were calculated within this subset.

MAFLD, metabolic dysfunction–associated fatty liver disease; BMI, body mass index; LDL-C, low-density lipoprotein cholesterol; HDL-C, high-density lipoprotein cholesterol; HbA1c, glycated hemoglobin; ALT, alanine aminotransferase; AST, aspartate aminotransferase.

In the multivariable logistic regression model in the total population in which sex, age, central obesity, hyperglycemia, elevated blood pressure, hypertriglyceridemia, and low HDL-C were entered simultaneously, all five MetS components were independently associated with higher odds of MAFLD (all *p* < 0.001) (Table 4). Central obesity showed the strongest association (OR 6.352, 95% CI 5.385–7.493), followed by hypertriglyceridemia (OR 3.979, 95% CI 3.508–4.513), elevated blood pressure (OR 2.041, 95% CI 1.775–2.347), hyperglycemia (OR 1.954, 95% CI 1.572–2.429), and low HDL-C (OR 1.543, 95% CI 1.236–1.927). Male sex (OR 7.488, 95% CI 6.361–8.816) and age (OR 1.009, 95% CI 1.004–1.014) were also independently associated with MAFLD. In terms of relative contribution to the model, sex had the largest Wald *χ*^2^ value (584.545), followed by central obesity (480.849), hypertriglyceridemia (460.925), and elevated blood pressure (100.468).

**Table 4:** Multivariable logistic regression of individual MetS components associated with MAFLD.

Predictor	Total population			Male			Female		
	Wald *χ*^2^	*p* value	OR (95%CI)	Wald *χ*^2^	*p* value	OR (95%CI)	Wald *χ*^2^	*p* value	OR (95%CI)
Sex(male vs. female)	584.545	<0.001	7.488 (6.361–8.816)						
Central obesity	480.849	<0.001	6.352 (5.385–7.493)	469.701	<0.001	6.544 (5.522–7.756)	23.919	<0.001	5.636 (2.818–11.269)
Hypertriglyceridemia	460.925	<0.001	3.979 (3.508–4.513)	283.691	<0.001	4.061 (3.450–4.780)	130.519	<0.001	3.310 (2.696–4.065)
Elevated blood pressure	100.468	<0.001	2.041 (1.775–2.347)	52.474	<0.001	1.968 (1.639–2.364)	29.652	<0.001	1.826 (1.470–2.269)
Hyperglycemia	36.451	<0.001	1.954 (1.572–2.429)	13.174	<0.001	1.690 (1.273–2.243)	34.504	<0.001	2.730 (1.953–3.817)
Low HDL-C	14.697	<0.001	1.543 (1.236–1.927)	5.608	0.018	1.341 (1.052–1.711)	9.377	0.002	2.303 (1.350–3.928)
Age	11.522	0.001	1.009 (1.004–1.014)	10.548	0.001	0.989 (0.982–0.996)	87.042	<0.001	1.042 (1.033–1.051)

Outcome was MAFLD (yes/no). Odds ratios (ORs) and 95% confidence intervals (CIs) were estimated using multivariable logistic regression with all predictors entered simultaneously. Analyses were performed in the total population and stratified by sex. MetS components were defined as follows: central obesity, waist circumference ≥90 cm in men or ≥85 cm in women; hyperglycemia, fasting plasma glucose ≥6.1 mmol/L or treated diabetes; elevated blood pressure, SBP ≥130 mmHg and/or DBP ≥85 mmHg or treated hypertension; hypertriglyceridemia, triglycerides ≥1.7 mmol/L; and low HDL-C, HDL-C < 1.04 mmol/L (16). Reference categories were female sex (in the total population model) and absence of each MetS component (in all models). Age was modeled as a continuous variable. The relative contribution of each variable to the model was assessed using the Wald *χ*^2^ statistic.

MAFLD, metabolic dysfunction–associated fatty liver disease; MetS, metabolic syndrome; SBP, systolic blood pressure; DBP, diastolic blood pressure; HDL-C, high-density lipoprotein cholesterol; OR, odds ratio; CI, confidence interval.

Sex-stratified analyses broadly confirmed the overall pattern observed in the total population, while also revealing differences in effect magnitude across sexes (Table 4). The prominent associations of central obesity and hypertriglyceridemia were generally preserved in both men and women: central obesity yielded ORs of 6.544 (95% CI 5.522–7.756) in men and 5.636 (95% CI 2.818–11.269) in women, while hypertriglyceridemia yielded ORs of 4.061 (95% CI 3.450–4.780) and 3.310 (95% CI 2.696–4.065), respectively. Elevated blood pressure showed moderate associations in both men and women, with ORs of 1.968 (95% CI 1.639–2.364) and 1.826 (95% CI 1.470–2.269), respectively. Hyperglycemia showed a larger association in women (OR 2.730, 95% CI 1.953–3.817) than in men (OR 1.690, 95% CI 1.273–2.243), and this sex difference was supported by a significant multiplicative interaction (*P* for interaction = 0.032; Supplementary Table S1). Low HDL-C also showed a numerically larger OR in women than in men, but the corresponding interaction with sex did not reach statistical significance (*P* for interaction = 0.071). Age showed opposite directions of association by sex, with a negative association in men (OR 0.989, 95% CI 0.982–0.996) and a positive association in women (OR 1.042, 95% CI 1.033–1.051), accompanied by a significant sex interaction (P for interaction < 0.001). Interactions between sex and central obesity, hypertriglyceridemia, or elevated blood pressure were not statistically significant. These findings indicate that, while all five MetS components were associated with MAFLD in both sexes, robust evidence of sex-related heterogeneity was mainly observed for hyperglycemia and age.

MAFLD prevalence increased markedly with increasing MetS component count (0/1/2/≥3 components: 14.04, 25.77, 55.76, and 79.68%, respectively; *P* for trend < 0.001). In the unadjusted model (Model 1), compared with participants with 0 components, the ORs (95% CIs) for MAFLD were 2.216 (1.769–2.554), 7.715 (6.366–9.350), and 23.996 (19.200–29.990) for 1, 2, and ≥3 components, respectively. After adjustment for age, sex, and BMI (Model 2), the corresponding ORs were 2.754 (2.224–3.410), 5.334 (4.243–6.705), and 10.264 (7.884–13.432). In Model 3, which additionally included VFA quartiles, the association remained significant, with ORs (95% CIs) of 2.558 (2.059–3.177), 4.784 (3.791–6.036), and 9.232 (7.025–12.132), respectively. VFA also showed an independent graded association with MAFLD, with ORs (95% CIs) of 1.894 (1.521–2.360), 2.440 (1.919–3.104), and 3.268 (2.436–4.386) for Q2, Q3, and Q4 versus Q1, respectively (Table 1).

Similar patterns were observed in the normal-BMI subgroup (BMI 18.5–23.9 kg/m^2^; *n* = 3,329). Compared with participants with 0 MetS components, the ORs (95% CIs) for MAFLD in Model 3 were 5.605 (3.590–8.751), 11.371 (7.018–18.424), and 21.269 (11.684–38.717) for 1, 2, and ≥3 components, respectively. VFA retained an independent graded association with MAFLD in this subgroup, with ORs (95% CIs) of 2.096 (1.521–2.889), 2.618 (1.753–3.912), and 3.668 (2.115–6.362) for Q2, Q3, and Q4 versus Q1, respectively (Table 2).

**Figure 2:**
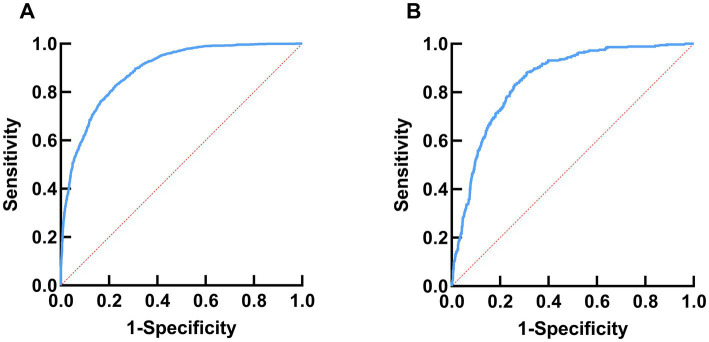
ROC curves for Model 3 predicting MAFLD. **(A)** Overall cohort. **(B)** Normal-BMI subgroup (BMI 18.5–23.9 kg/m^2^). Model 3 incorporates MetS component count, VFA quartiles, age, sex, and BMI. AUC values (95% CI): overall cohort 0.887 (0.879–0.894), normal-BMI subgroup 0.849 (0.830–0.867); both *p* < 0.001 for the test of AUC > 0.5.

The fully adjusted model incorporating MetS component count and VFA showed favorable discriminatory performance for MAFLD in the overall population, with an AUC of 0.887 (95% CI 0.879–0.894) (Figure 2A). This performance was largely retained in the normal-BMI subgroup, with an AUC of 0.849 (95% CI 0.830–0.867) (Figure 2B). In supplementary ROC analyses, the corresponding AUCs for models combining MetS component count with continuous VFA, WC, or WHR in the overall population were 0.887 (95% CI 0.879–0.895), 0.887 (95% CI 0.880–0.895), and 0.887 (95% CI 0.879–0.894), respectively (Supplementary Table S2). In the normal-BMI subgroup, VFA, WC, and WHR each showed fair discriminatory ability for MAFLD when modeled as continuous variables after adjustment for age, sex, and BMI, with AUCs of 0.778 (95% CI 0.756–0.800), 0.779 (95% CI 0.756–0.802), and 0.767 (95% CI 0.743–0.791), respectively. Pairwise comparisons using the DeLong test showed no significant differences among these three AUCs (VFA vs. WC: *p* = 0.921; VFA vs. WHR: *p* = 0.283; WC vs. WHR: *p* = 0.316) (Supplementary Table S3).

## Discussion

In this single-center health-checkup cohort, we comprehensively evaluated the associations of MetS component count and visceral fat area (VFA) with MAFLD. We found a pronounced dose–response pattern, with MAFLD prevalence increasing stepwise across higher MetS component count categories. This association remained robust after sequential adjustment for age, sex, and BMI, indicating that cumulative metabolic burden may better reflect MAFLD risk than reliance on BMI or any single metabolic marker alone. In addition, VFA showed an independent graded association with MAFLD, underscoring the relevance of visceral adiposity in MAFLD-oriented risk assessment. These patterns were also observed in the normal-BMI subgroup, and the fully adjusted model showed good discrimination in both the overall population and the normal-BMI subgroup.

The overall prevalence of MAFLD in this cohort was 38.8% (2,794/7,192) and was significantly higher in men than in women (52.7% [1,968/3,734] vs. 23.9% [826/3,458]; *p* < 0.001), consistent with findings reported by Huang et al. (20). In keeping with this pattern, compared with non-MAFLD participants, those with MAFLD showed a more adverse cardiometabolic profile, including greater central and visceral adiposity, higher blood pressure, higher triglycerides, higher fasting glucose and HbA1c, and lower HDL-C. These findings are consistent with the concept that MAFLD represents a hepatic manifestation of systemic metabolic dysregulation, in line with prior reports (21, 22). Taken together, our findings suggest that the cumulative number of MetS components may provide a more informative basis for MAFLD-oriented risk assessment than isolated metabolic markers or BMI alone.

Prior studies have consistently linked metabolic syndrome and visceral adiposity to the development and progression of MAFLD, yet the mechanistic pathways remain incompletely defined (23–26). A plausible explanation centers on insulin resistance and impaired lipid handling as core pathophysiologic processes in MAFLD: increasing insulin resistance amplifies lipotoxicity, glucotoxicity, and chronic low-grade inflammation, thereby promoting hepatic steatosis and fibrogenic remodeling (27). In this framework, higher MetS component count can be viewed as a pragmatic surrogate for the cumulative severity of systemic insulin resistance and adipose tissue dysfunction (28). Moreover, MetS component count likely reflects concurrent exposures to multiple inflammatory and oxidative stress signals—emanating from adipose dysfunction, hyperglycemia-related pathways, and gut–liver axis perturbations—which may act in concert to promote hepatic steatosis and metabolic liver injury (29, 30). In particular, gut–liver axis dysregulation may promote hepatic lipid accumulation and inflammatory signaling through increased intestinal permeability, microbial-derived metabolites, and endotoxin-mediated activation of hepatic immune pathways, thereby linking systemic metabolic dysfunction with liver injury (31). Dysfunctional visceral adipose tissue may further exacerbate hepatic metabolic stress by releasing excess free fatty acids and pro-inflammatory adipokines, with portal delivery of free fatty acids to the liver directly favoring intrahepatic triglyceride accumulation (32). Consistent with this biology, VFA exhibited an independent graded association with MAFLD in our study (Q4 vs. Q1: OR 3.268, 95% CI 2.436–4.386), underscoring visceral adiposity as a key correlate beyond general adiposity and aligning with prior evidence (33).

We also observed sex-related heterogeneity in the associations of individual MetS components with MAFLD. Although all five MetS components remained significantly associated with MAFLD in both sexes, formal interaction analyses indicated that robust sex-related heterogeneity was mainly evident for hyperglycemia and age, suggesting that the metabolic correlates of MAFLD may not be entirely uniform across sexes. Of note, the higher overall MAFLD risk observed in men does not conflict with the stronger relative association of hyperglycemia in women; the former reflects an overall between-sex difference in MAFLD burden, whereas the latter indicates component-specific sex-related heterogeneity supported by formal interaction analysis (P for interaction = 0.032, Supplementary Table S1). The stronger association of hyperglycemia with MAFLD in women may reflect sex-specific differences in body fat distribution, hormonal milieu, and susceptibility to glycometabolic dysfunction (34). In addition, the divergent age associations between men and women may partly reflect age-related hormonal and metabolic changes in women, particularly around the menopausal transition (35), as well as differences in the age-related distribution of metabolic risk among men in this health-checkup population.

In the normal BMI subgroup, higher MetS component count remained strongly associated with MAFLD, and VFA also retained an independent graded association. These findings suggest that individuals with normal-weight obesity (NWO) or metabolically obese normal weight (MONW) may represent an overlooked high-risk population that could be missed by BMI-based screening alone. Moreover, differences in MetS component count and VFA may help explain why individuals with similar BMIs can exhibit markedly different risks of MAFLD. Visceral adipose tissue is closely linked to multiple components of MetS, and its excessive accumulation is considered a central driver of metabolic syndrome progression (36). By releasing excess free fatty acids and pro-inflammatory adipokines, visceral fat may contribute to insulin resistance and hepatic metabolic injury (29, 37). Taken together, these findings support the assessment of adiposity-related indicators beyond BMI in apparently normal-weight individuals to improve recognition of hidden metabolic risk and MAFLD.

The fully adjusted model incorporating MetS component count and VFA showed good discrimination for MAFLD in the overall population (AUC 0.887, 95% CI 0.879–0.894) and in the normal-BMI subgroup (AUC 0.849, 95% CI 0.830–0.867). In supplementary analyses, models combining MetS component count with continuous VFA, WC, or WHR yielded nearly identical AUC values in the overall population. In the normal-BMI subgroup, VFA, WC, and WHR each showed fair discriminatory ability, and pairwise comparisons revealed no significant differences among their AUCs. Taken together, these findings suggest that combining cumulative metabolic burden with adiposity-related indicators may support MAFLD-oriented risk assessment in routine health screening, while also indicating that the discriminatory value of VFA should be interpreted alongside that of simpler anthropometric measures rather than assumed to be clearly superior.

### Limitations

This study has several limitations. First, the cross-sectional design precludes causal inference, and the temporal relationships among MetS component accumulation, visceral adiposity, and MAFLD could not be determined. Second, although major demographic and metabolic factors were adjusted for, residual confounding from unmeasured lifestyle-related variables, such as diet, physical activity, and smoking, may remain. Third, hepatic steatosis was assessed by ultrasonography and VFA by bioelectrical impedance analysis. Although ultrasonography is widely used in population-based screening and has acceptable diagnostic performance for fatty liver, it remains a qualitative rather than quantitative method and may be less sensitive for mild steatosis (38, 39). Similarly, although BIA-derived VFA has shown acceptable agreement with CT- or MRI-based measurements in previous studies, some measurement error is still possible (40, 41). Fourth, this was a single-center study of a health-checkup population, which may limit the generalizability of the findings to other clinical or community-based populations. Finally, because the present study was designed primarily to evaluate associations rather than to develop a parsimonious prediction model, we did not further assess whether a reduced set of the highest-ranking variables could reproduce the discriminatory performance of models incorporating all five MetS components. Future studies should develop and externally validate simplified MAFLD-oriented risk models in independent cohorts.

## Conclusion

In conclusion, this study demonstrated a clear dose–response association between MetS component count with MAFLD, and showed that VFA was independently associated with MAFLD. These findings support the value of integrating cumulative metabolic burden and adiposity-related indicators into MAFLD-oriented risk assessment in health-checkup populations. Future longitudinal or repeated cohort studies are warranted to validate the predictive value of MetS component count and VFA for the incidence, progression, and fibrosis risk of MAFLD. Such studies may also help determine how these easily obtainable metabolic and adiposity-related indicators can be incorporated into practical screening pathways. In addition, further studies are needed to explore the integration of MetS component count and adiposity-related indicators with non-invasive fibrosis scoring systems or elastography-based tools to support more personalized risk stratification and management of MAFLD.

## Data Availability

The data analyzed in this study is subject to the following licenses/restrictions: the datasets generated and/or analyzed during the current study are available from the corresponding author upon reasonable request. Requests to access these datasets should be directed to jcm312@tmmu.edu.cn.
