# Supplementary material for: Dose–response association between metabolic syndrome component count and metabolic dysfunction-associated fatty liver disease, and independent graded association of visceral fat area: a cross-sectional study in a health-checkup population
Source: Front Public Health. 2026 Jun 16;14:1842607. doi: 10.3389/fpubh.2026.1842607 (PMC13314849; doi:10.3389/fpubh.2026.1842607)
Supplement: Supplementary file 1 [file Table_1.docx]

# Supplementary Table S1

**Supplementary Table S1.** Interaction effects between sex and each metabolic syndrome component on MAFLD risk

| **Interaction term** | **P for interaction** | **OR (95% CI)<sup>*****</sup>** |
| --- | --- | --- |
| Sex × Central obesity | 0.681 | 0.861 (0.422–1.758) |
| Sex × Hypertriglyceridemia | 0.127 | 0.815 (0.627–1.060) |
| Sex × Elevated blood pressure | 0.605 | 0.928 (0.699–1.232) |
| Sex × Hyperglycemia | **0.032** | 1.615 (1.042–2.505) |
| Sex × Low HDL‑C | 0.071 | 1.717 (0.955–3.087) |
| Sex × Age | **<0.001** | 1.054 (1.042–1.065) |

* The OR for each interaction term represents the multiplicative modification by sex of the association between the corresponding MetS component or age and MAFLD risk. All interaction terms were entered simultaneously into a multivariable logistic regression model adjusted for all main effects (sex, age, and all MetS components).

P values were derived from Wald χ² tests. Statistically significant interactions (P < 0.05) are highlighted in bold.

MAFLD, metabolic dysfunction‑associated fatty liver disease; MetS, metabolic syndrome; HDL‑C, high‑density lipoprotein cholesterol; OR, odds ratio; CI, confidence interval.
