# Supplementary material for: Dose–response association between metabolic syndrome component count and metabolic dysfunction-associated fatty liver disease, and independent graded association of visceral fat area: a cross-sectional study in a health-checkup population
Source: Front Public Health. 2026 Jun 16;14:1842607. doi: 10.3389/fpubh.2026.1842607 (PMC13314849; doi:10.3389/fpubh.2026.1842607)
Supplement: Supplementary file 2 [file Table_2.docx]

# Supplementary Table S2

**Supplementary Table S2.** Odds ratios and AUC for MAFLD predicted by MetS component count in combination with VFA, WC, or WHR, respectively

| **Predictor** | MetS component count +VFA, OR (95% CI) | MetS component count +WC, OR (95% CI) | MetS component count +WHR, OR (95% CI) |
| --- | --- | --- | --- |
| MetS component count (ref = 0) |  |  |  |
| 1 component | 2.599(2.098-3.220) | 2.452(1.977-3.041) | 2.601(2.098-3.225) |
| 2 components | 4.968(3.949-6.250) | 4.370(3.459-5.521) | 4.783(3.795-6.028) |
| ≥3 components | 9.605(7.328-12.589) | 7.644(5.787-10.096) | 8.992(6.853-11.798) |
| VFA ( cm²) | 1.014(1.010-1.018) | - | - |
| WC ( cm ) | - | 1.056(1.042-1.070) | - |
| WHR( per 0.1-unit increase ) | - | - | 1.690(1.458-1.958) |
|  |  |  |  |
| AUC | 0.887 | 0.887 | 0.887 |
| 95%CI | 0.879-0.895 | 0.880-0.895 | 0.879-0.894 |
| P for AUC > 0.5* | <0.001 | <0.001 | <0.001 |

Outcome was MAFLD. Each model included MetS component count and one adiposity-related indicator (VFA, WC, or WHR), with adjustment for age (continuous), sex, and BMI. VFA, WC, and WHR were entered as continuous variables, and the reference category for MetS component count was 0 components. The ORs and 95% CIs for WHR corresponded to a 0.1-unit increase, derived by exponentiating the original regression coefficient (B = 5.246, SE = 0.751) multiplied by 0.1; the original model was fitted using raw WHR values, and the P value remained unchanged. ^*^P value for the null hypothesis that AUC = 0.5.

MAFLD, metabolic dysfunction-associated fatty liver disease; MetS, metabolic syndrome; VFA, visceral fat area; WC, waist circumference; WHR, waist-to-hip ratio; AUC, area under the ROC curve; CI, confidence interval.
