# Supplementary material for: Dose–response association between metabolic syndrome component count and metabolic dysfunction-associated fatty liver disease, and independent graded association of visceral fat area: a cross-sectional study in a health-checkup population
Source: Front Public Health. 2026 Jun 16;14:1842607. doi: 10.3389/fpubh.2026.1842607 (PMC13314849; doi:10.3389/fpubh.2026.1842607)
Supplement: Supplementary file 3 [file Table_3.docx]

# Supplementary Table S3

**Supplementary Table S3.** AUC of VFA, WC, and WHR as continuous variables for predicting MAFLD in the normal‑BMI subgroup

| **Model (adjusted for sex, age, BMI)** | **AUC** | **95%CI** | **P for AUC > 0.5*** |
| --- | --- | --- | --- |
| VFA (cm²) | 0.778 | 0.756-0.800 | <0.001 |
| WC (cm) | 0.779 | 0.756-0.802 | <0.001 |
| WHR | 0.767 | 0.743-0.791 | <0.001 |

Outcome was MAFLD in the normal-BMI subgroup (BMI 18.5–23.9 kg/m², defined according to Chinese criteria for adults). Each model was adjusted for age (continuous), sex, and BMI. VFA, WC, and WHR were entered as continuous variables.

*P value for the null hypothesis that AUC = 0.5.

Pairwise comparisons using DeLong’s test showed no significant differences among the three AUCs (VFA vs. WC: P = 0.921; VFA vs. WHR: P = 0.283; WC vs. WHR: P = 0.316).

MAFLD, metabolic dysfunction–associated fatty liver disease; BMI, body mass index; VFA, visceral fat area; WC, waist circumference; WHR, waist-to-hip ratio; CI, confidence interval.
